# Employment status and differences in the one-year coverage of physician visits: different needs or unequal access to services?

**DOI:** 10.1186/1472-6963-6-123

**Published:** 2006-10-02

**Authors:** Pekka Virtanen, Mika Kivimäki, Jussi Vahtera, Markku Koskenvuo

**Affiliations:** 1 School of Public Health, University of Tampere, Tampere, Finland; 2 Department of General Practice, Pirkanmaa Hospital District, Tampere, Finland; 3 Department of Psychology, University of Helsinki, Helsinki, Finland; 4 The Finnish Institute of Occupational Health, Helsinki, Finland; 5 The Finnish Institute of Occupational Health, Turku, Finland; 6 Department of Public Health, University of Helsinki, Helsinki, Finland

## Abstract

**Background:**

The dichotomy employed vs. unemployed is still a relevant, but rather crude measure of status in current labour markets. Also, studies concerning the association of employment status with health have to specify the type of the employment as well as the characteristics of the unemployment. This study aims to reveal differences and potential inequalities in physician visits among seven groups in the core-periphery structures of the labour markets.

**Methods:**

A total of 16 000 Finns responded to a postal survey in 2003. Their visits to physicians in public primary health care, occupational health care, private health services, hospital outpatient clinics and dental care services during previous year were measured as indicators of service utilisation. Participants were classified as employees having a permanent or fixed-term and full-time or part-time contract and as those experiencing short-term, prolonged or long-term unemployment. Differences in the one-year coverage of physician visits between these groups of employees were analysed using logistic regression analyses where differences in the need for services were controlled for by including demographics and self-rated health assessments in the models.

**Results:**

Permanently employed respondents had visited a physician most often, and the need-adjusted regression models showed significantly lower odds ratios for a visit among fixed-term employees (OR 0.65, 95% CI 0.53–0.81) and in particular among the long-term unemployed (OR 0.21, 95% CI 0.14–0.31). A stratified analysis according to health care sector showed the lowest odds ratios in occupational health care and private physicians (ORs between 0.05 and 0.73) and also low odds ratios for dentists (ORs between 0.45 and 0.91), whereas visits to public primary health care were more common among non-permanent employees and the unemployed (ORs between 1.46 and 2.39).

**Conclusion:**

The use of physician services varies according to labour market status, being relatively low among the non-permanently employed and the unemployed. This underuse is emphasised when clinical need is taken into account. The main reasons for the variance evidently lie in the structures of the Finnish health service system. The result may indicate non-optimal health care of the population on the periphery of the labour market, but it may also reflect the importance of employment status as a context for need and the decision to visit a physician.

## Background

Differences in the use of health services may reflect differences in health and clinical needs, but they may also reflect differences in access to these services. In terms of health policy, the identification of population level differences in access to health services is particularly important in that it indicates groups towards whom interventions should be targeted. The present study examines the use of health services with the aim of revealing potential inequalities in access among population groups defined by their labour market status.

Research into the associations of the major 'atypical' status – in other words unemployment – with health service contacts has a long tradition. One series of studies consists of follow-ups centred around factory closures. In a Danish study [1] hospital admissions decreased among the group studied and increased in the permanently employed control group after the closure of a shipyard. On the other hand, a factory closure in England led to increased visits to general practitioners [2]. In Austria, Studnicka et al. [3] also found increased utilisation of services, such as self-treatment, physician visits or hospital care, in those who were unemployed one year after the closure. However, the association turned out to be non-significant when adjusted for both psychological and physical health. A Finnish follow-up study of the long-term unemployed demonstrated a higher visit rate to primary health care services during a six month re-employment period than during the period of unemployment that preceded this [4].

Another series of studies consists of comparisons between unemployed and employed populations. An Austrian survey [5] found that the rate of GP consultations during the previous year was higher among the unemployed than among employed respondents, and specialist consultations were more frequent among unemployed women. In Canada the unemployed used more physicians' services both during the 1970s (as measured in visits and telephone consultations [6]) and during the 1980s (as measured from records of in-patient hospital admissions and ambulatory physician contacts [7]). According to a British study [8] unemployed men consulted general practitioners more often, even after adjustment for self-reported longstanding illness. In Finland the unemployed visited public primary health care more often, but private physicians, occupational health care and dental care less often [9].

Most of the research reviewed above is based on data from the 1970s and the 1980s. The results may be outdated because of changes that have taken place during the 1990s in health care financing and delivery arrangements. These changes may have facilitated or created obstacles to the unemployed regarding entry to health services. Their access to these services may be less easy as a result of exclusion from employment-related insurance systems, lower levels of income or lack of workplace-based occupational health care. On the other hand, new employment policy measures may include novel ways of providing health services to the unemployed.

A second and potentially more important reason for repeating and refining the research on employment status and the use of health services are the changes that have taken place in the labour markets of Western countries since the 1980s. Increased flexibility [10-12] has led to more diverse employment arrangements, and the spectrum of unemployment ranges from a 'buffer work force' with underemployment and occasional unemployment episodes to a 'hard core' of long-term unemployed with poor prospects of re-employment on the open labour market. The traditional dichotomy of employed versus unemployed is still relevant, but it is rather crude and may mask potential differences in service use. A more detailed specification and breakdown of the core-periphery structure of the labour market is necessary when studying the relationships of employment status with health [13] and, in addition, the use of and access to health services.

Thirdly, there is need to explicate the dual nature of service use. A study should distinguish between clientship as an indicator of a health problem and clientship as an indicator of features of a particular health care system. The problems in measuring differences and changes in health status using health care contacts were emphasised by Kasl, Gore & Kobb back in 1975 with their seminal study of factory closure [14]. They found no consistent associations between the phases of job loss and the re-employment process and the number of days on which a doctor was consulted, thus concluding that, in the absence of serious medical difficulties, this measure seemed to be sensitive to aspects of the social environment, while the relationship to perceptions of health was rather weak. The complexity of individuals' decisions to seek care and the pathways by which they arrive at medical facilities were also emphasised in Bartley and Fagin's [15] comments on the results of Danish shipyard workers' hospital admissions [1]. In accordance with this view, Studnicka & Schreiber [5] interpreted their findings, regarding the absence of differences in the utilisation of health services between the employed and the unemployed, in the contexts of disturbed doctor-patient relations and the changed significance of the sick role during unemployment.

The theoretical framework and methodology of this study was adopted from research on socioeconomic health inequalities. Quantitative description of differences between population groups implies that the economic and political structures of a society create social divisions, classes, hierarchies, antagonisms and conflicts that produce and reproduce health inequalities. According to radical versions of conflict theory, these elements of domination and repression are also inherent in the health service systems. However, while assuming that the policies of the welfare state contribute to health inequalities, conflict theory in fact assumes that the regime in general and the health services in particular can be organised with an eye to reducing inequalities. For this reason it is important to note the country of a particular study, as we have done above. Inconsistent results in the cited research may be due, at least partly, to differences in health service systems between nations.

The patterns of health service use among various non-permanent employees and among subgroups of the unemployed have been hitherto neglected in health service research, in spite of the fact that studies have linked health differences to labour market status [13]. In this study we aim to assess the extent to which the differences in the use of health services among permanent employees, fixed-term employees, part-time employees, the short-term unemployed and the long-term unemployed are not explained by variations in medical needs or by the needs associated with other socio-demographic characteristics.

## Methods

### The setting of the study: Finland's health care system and labour market

Finnish public primary health care is provided by municipal health centres. There is also a relatively large private sector, as well as a legislation-based occupational health service system which is, when compared internationally, exceptionally large, covering most employed citizens. Finland's working age population (about 3.3 million) make about 10 million primary health care visits per year, about half of which take place in health centres, one quarter in occupational health care and one quarter in the private sector. Occupational health services are free, whereas fixed fees are charged for visits to health centres, and in the private sector, the prices are market dependent with fixed compensation provided by universal sickness insurance. Moreover, about 4 million visits take place in hospital outpatient clinics. Dental services are offered by municipal health centres, but the supply is insufficient, and the use of private dentists is common.

The income-related inequality in the use of physicians' services, both in terms of GP visits and specialist visits, is in Finland the highest in Europe [16]. The reasons for this lie in the structures and financing of the service organisation as outlined above. At the same time, there are large inequalities in the distribution of labour. The internationally high unemployment figures have their roots in the exceptionally deep depression that hit Finland at the beginning of the 1990s, and the proportion of fixed-term employees in one of the highest among the EU countries. These features of Finnish health care and Finnish labour markets provide good reason for detailed studies concerning the use of ambulatory health services in relation to employment status.

### Participants

The Health and Social Support Project (HeSSup) is a longitudinal study on a population sample representative of the Finnish population of four age groups: 20–24, 30–34, 40–44 and 50–54. The study was launched in 1998 with a postal questionnaire that yielded, with a response rate of 40%, 21 101 participants [13]. The second phase questionnaire was posted in 2003 to those participants who could be found in the population register (n = 19950). The present study is based on the responses obtained from this follow-up survey (n = 16000, response rate of the eligible cohort 80.2%). After excluding respondents who were not working or seeking a job such as students, retired people, or housewives (n = 3 373), the employed participants were classified into those having a permanent or a fixed-term employment contract and those working full-time or part-time (less than 20 hours weekly), while the unemployed respondents were classified into those receiving earnings-related compensation which is paid for the first 500 unemployment days (short-term unemployment) and those receiving the basic compensation paid after 500 days. The last group was further split on the basis of the employment status they reported during the first phase survey in 1998: the situation of participants who had received unemployment compensation was defined as long-term unemployment, while the situation of those who had not received compensation five years ago was defined as prolonged unemployment.

### Measures

The survey included a question inquiring whether respondents had visited – "because of illness, symptoms or other problems" – a physician in a public health centre, occupational health care, the private sector, a hospital outpatient clinic or in dental services during the previous twelve months. Answering options were no visits, 1 visit, 2–4 visits or over 4 visits. Utilisation of the services was expressed as coverage, or as a percentage of respondents with one or more visits.

In order to show potential inequalities in service utilisation it is necessary to control for actual health and consequent 'medical need' of health care [17]. There are several ways to assess this need, but the most commonly used are perceived health and reported illnesses [17-20]. In this study perceived health was measured with the five-class variable of self-rated health (good, fairly good, average, fairly poor or poor), the occurrence of breathlessness (yes/no according to the NYHA classification), and also according to the incidence of depression (yes/no as assessed with the 21-item version of Beck's Depression Inventory [21] using a score of 10 as the cut-off point). Diseases diagnosed by a physician were measured with a checklist (yes/no) of common conditions including cardiovascular problems (hypertension, angina, myocardial infarction or stroke), respiratory ailments (asthma or chronic obstructive bronchitis), musculoskeletal diseases (sciatica, rheumatoid arthritis, osteoarthritis, fibromyalgia), mental disorders (depression, panic disorder or some other mental illness), and other diseases (diabetes, coeliac disease, liver disease, renal disease, epilepsy, other neurological disease, cancer, other severe disease).

### Data analysis

We chose permanent full-time employees as the reference group and used binary logistic regression analyses to obtain odds ratios for at least one physician visit according to employment status. Perceived health and reported diseases (dichotomous variables measuring any disease, cardiorespiratory disease and musculoskeletal disease) were used to control for medical need. Gender, age and level of education (no vocational education, vocational school, college, university) were controlled for as background factors.

## Results

The characteristics of the respondents are presented in Table 1. There were more women than men among the employed, in particular among part-time and fixed-term employees. Fixed-term full-time employees were the youngest. Long-term unemployment was more common among men. As expected, the unemployed were poorly educated and their health was poor. More than half of the short-term unemployed were in the 55–59 age group. The reason for this is the unemployment pension, in other words the earnings-related compensation with unlimited duration, to which this age group is entitled. Thus, the group consisted partly of 'voluntary long-term unemployed' and only partly of short-term unemployed who were genuinely seeking work. Because this 'contamination' of the group might have biased the results, it was excluded from subsequent analyses.

The coverage of services and the number of visits, in other words, the intensity of the service use, may be partly determined by different factors [22]. Analysis by the number of visits (Table 2) suggests, however, that differences in the coverage by labour market status are reflected in corresponding differences in annual intensity of physician visits.

Table 3 shows that permanent employees had visited a physician more often than fixed-term employees and markedly more often than the unemployed. These differences widened after adjustment for medical need, in particular when perceived health was used as an indicator of need. For example, the odds ratio for a visit was 4 times lower (odds ratio 0.25) for the long-term unemployed than for employees with a full-time permanent contract. The absence of significant interactions suggests that these findings are not dependent on age, gender or education. Further analysis, which separated respondents without disease from those with disease (Table 4), showed that the coverage differences were similar in both subgroups among the employed respondents and among the long-term unemployed, whereas the differences were smaller among those having a disease combined with short-term or prolonged unemployment. Even in these cases, however, the odds ratios were under 1, and according to the interaction test, the difference between the subgroups with and without illness was non-significant (p-value 0.422).

Table 5 presents the association between labour market status and coverage of the services according to sectors of health care. After adjustment for medical need (as measured with perceived health), the odds ratios of a visit to public primary health care were 1.4–1.8 times higher for non-permanent employees than for full-time permanent employees, and the corresponding odds ratios for the unemployed were 1.8–2.4. As might be expected, the probability of visits to occupational health care was lower the more peripheral the labour market status, and a similar although less pronounced pattern was seen in the use of private physicians' services. Visits to hospital outpatient clinics tended to be more common among the unemployed, but the differences were non-significant in all groups. Odds ratios for visits to dentists were generally low, and significant marginalisation from dental health care was seen among fixed-term employees and among the long-term unemployed.

Finally, we focused the analysis on respondents who reported a cardiovascular and/or respiratory disease and on those who reported a musculoskeletal disease, and concentrated on the coverage of visits to physicians in primary health care (i.e. in public health centre and occupational health care and private services) and to hospital outpatient clinics (i.e. to specialised secondary health care). Except for the group with prolonged unemployment and musculoskeletal disease, the odds ratios revealed that the non-permanent employees and the unemployed visit primary health care less often, while there is a tendency towards more visits to hospital outpatient clinics (Table 6).

## Discussion

Our study aimed to seek potential differences in the use of ambulatory physician services between people in different employment situations. The higher coverage of services among permanent full-time employees compared with other employees and the unemployed could not be attributed to needs arising from medical conditions or health impairments; on the contrary, the analyses showed a greater inequality between this group and the people occupying less stable labour market status when adjusted for clinical need. According to the analysis by service sector, permanent employees visited workplace physicians and private physicians more frequently, whereas fixed-term employees and the unemployed used public GP services. Corresponding differences were present but less pronounced in dental care. With ambulatory hospital visits no differences between employment statuses were found.

### Reasons for inequality

Our results are in line with previous Finnish studies showing an increase in visits to physicians after re-employment [4] and a higher frequency of visits to public primary health care among the unemployed [9]. Although previous studies (e.g. [23]) have reported socioeconomic inequality in Finnish hospital care, we found no evidence of this in ambulatory hospital visits. However, these studies concern quantity and quality of inpatient care, and our results, in fact, do not contradict their findings.

One explanation for our results is evidently the more comprehensive health service spectrum available to permanent full-time employees: in addition to GPs (in public health centres) they commonly have access to the occupational health care physician of the workplace, and they can also afford to visit various specialists (including dentists) in the private sector. From the perspective of the individual client the services certainly have additional value, but from the perspective of society the health care serving permanent employees may involve inappropriate and uneconomic overlapping.

The results concerning participants with a diagnosed disease also prompt the question of whether the coverage differences indicate marginalisation of the unemployed and fixed-term employees or medically unnecessary visits to physicians among permanent employees which may be related to employment. If we assume that absence of disease equals medical need of services the figures of Table 4 support the latter conclusion. If we assume that presence of disease equals the need, the former conclusion also gets support, in particular as regards fixed-term employees and the long-term unemployed. Thus, the answer would be 'both'. The reasoning is, however, complicated by the possibility of reverse causality, in other words, one cannot have a 'disease diagnosed by a physician' without visiting a physician, and the disease reported in the questionnaire may in fact be a consequence of the visit. Reverse causality might explain why adjustment for perceived health in the analyses of Table 3 lowers the odds ratios more than adjustment for disease.

The analysis of participants with cardiorespiratory and musculoskeletal problems serves to develop the discussion. According to national Current Care Guidelines, people with a chronic disease – in particular a cardiovascular or respiratory disease – should visit a physician at least once a year. No visit during twelve months therefore indicates obvious underuse of health services, or marginalisation from clinically relevant care. Table 6 illustrates that this is the case with fixed-term employees as well as with the unemployed. Moreover, the figures show that visits to health centres only partially compensate the inequality in use of primary health care created by occupational health and private services. The relatively high frequency of hospital outpatient visits made by these groups may also be interpreted as a compensatory action, but it may also indicate the need for more frequent specialist consultations because of a more severe condition. Finally, the conclusions remain uncertain since we do not know whether the visits had been made due to reported diseases or some other health problem.

In all, the study found considerable differences in the use of ambulatory physician services between people with different labour market statuses, and these differences grew when the mismatch between health status and use of services was taken into account. In particular, the health care of the long-term unemployed seems to be inadequate.

In the Finnish health service system, fixed-term employees and unemployed people seem to end up as clients of public health centres rather than any other sectors. The GPs in public health centres have received training to meet the needs of various client groups, but the question can still be raised as to whether they are aware of the specificity of their working-age clientele, whether they are competent, and whether postgraduate medical education should be available for handling the health problems of patients in more or less 'atypical' career situations.

The results may also reflect the importance of labour market status as a non-medical factor influencing health service need. It is evident that the care-seeking decisions of the unemployed are affected by less immediate needs to justify the sick role. In the case of a fixed-term or a part-time employee there may be reluctance to adopt the sick role, as it may risk the job contract or future career. The frequency and the nature of medical consultations may also differ due to differences in clinical needs such as symptom alleviation and rehabilitation. The need to optimise physical and mental fitness is probably more urgent in working life than in the everyday life of an unemployed individual, and feelings of hopelessness and indolence may affect the priority and perception of seeking health care. Moreover, an unemployed individual who feels ill may be reluctant to consult a physician for fear that the moral connotations of the employment situation become explicit, or for fear that a document of poor health may have an unfavourable influence on employment prospects. Correspondingly, non-permanent employees may perceive increased job insecurity to be a consequence of seeking health care.

The Finnish practice of sickness absence certification generates a need for 'clinically unnecessary' visits, in particular among permanent employees, who have more sickness absences than non-permanent ones [24]. The unemployed are also expected to present a certificate for being 'absent from the dole' when ill, although they seldom do this and, then, only in cases of longstanding disability.

### Sociological viewpoints

Certification of sickness absence is a concrete example of health care functioning as an institution of social control [25]. As a part of society, health care, and in particular occupational health care, is bound to serve society's dominant ideas and values. In Western capitalist societies the comprehensive health services of the permanently employed core work force may be interpreted not only as benevolent promotion of their wellbeing, but also as exploitative maintenance of their production capacity. Correspondingly, the buffer work force and the unemployed could be seen as marginalised from these services because their contribution to the production – and consumption – of commodities is less important. On the other hand, in an employment society unemployment is perceived as deviance, but for an individual it also means 'freedom' from work. Indeed, the present findings are consistent with the possibility that unemployment may free an individual from unnecessary medicalisation and the domination associated with it, as well as iatrogenic health problems.

As well as viewing the relationship of medical encounter with labour market status in terms of conflict theory, it can be considered through a number of less macrosociological and less structural frameworks. The analogy of the sick role with the role of the unemployed is obvious. An unemployed citizen has also failed to comply with social expectations, is dysfunctional for the social system, and needs to be controlled and regulated. The role of unemployed legitimates withdrawal from a social obligation – in other words work – and the unemployed individual is exempted from responsibility, that is to say (s)he is not blamed for his/her inability to keep or get a job. However, these rights are granted only on condition that (s)he shows motivation and co-operates in getting re-employed. (S)he is required to utilise relevant employment services and enrol as a job seeker at the labour force bureau. It is evident that illness and unemployment, when occurring simultaneously, have a new significance for an individual, but we may also ask whether the roles are partly interchangeable, in particular in the case of chronic illness and/or unemployment. Thus, we may ask whether health services and employment policy services are separate or partly alternative social systems for adapting deviant individuals. Furthermore, 'double deviance' on the part of the client may impact on the practices of professionals providing health and employment services.

The above analogy is by no means specific to the Parsonian, structural-functionalist framework. Utilising the Foucauldian concepts [26] health as well as employment services are constituents of the 'panopticon', or the set of social institutions used for the surveillance of citizens and for the execution of expertise and professional power. Indeed, the relative marginalisation of the unemployed from the health services may partly indicate that they tend to avoid medicalisation of their problems, and that they also have more freedom with regard to the sick role and to the social control and surveillance carried out by the health care institutions.

### Methodology

Physician consultation is both quantitatively and qualitatively the most important contact between an ill citizen and the health care system. There are also contacts with nurses, physiotherapists, psychotherapists and other health professionals, as well as with unofficial service providers, but it is unlikely that differences in the use of these services compensated the differences observed in this study. Rather, nurse contacts are most common in occupational health care, and there are also a large number of physiotherapists.

The investigation regarding visits to physicians in specified organisations may omit some contacts such as those taking place in student health services and military health care, but the vast majority of these groups were excluded from the study. Thus, the rate of visits to physicians used in this study may be considered a valid measure for the use of and access to health care.

Studies show that respondents remember recent visits to physicians fairly well, and that there is no association between demographic or health variables and the tendency towards discrepancy between self reported and registered visit rates [27].

With a response rate of 80% it is reasonable to assume that the respondents represent the population recruited in the HeSSup study and the follow-up. The low participation at baseline is analysed in detail elsewhere [28]: the unemployment rate of the participants differed significantly from the expected (8.6% vs. 9.3%, odds ratio 0.92, confidence interval 0.88–0.97), but in the light of absolute figures this, as well as the differences in sex, marital status, education and indicators of health, could be considered as acceptable. Moreover, the proportion of unemployed respondents (6.9%) corresponded fairly well to the falling national unemployment figures [29] at the time of the second phase survey. Thus, although we cannot be sure that the respondents were a representative sample of the Finnish population, we may reasonably conclude that the sample was not too biased from the viewpoint of our study questions.

Smaje and Le Grand [17] present an in-depth discussion of the factors affecting the utilisation of health services, in particular of medical need as a confounding factor in comparisons of utilisation among population groups. The multivariate analysis applied in this study is similar to Smaje and Le Grand's model with the exception that, instead of ethnicity, our grouping was based on labour market status. We controlled separately for perceived health and reported disease as factors affecting service need. The results lend support to earlier evidence that people may be more likely to report long-term illness if they have recently visited a physician [30]. Therefore, adjustment merely for reported disease as a measure of medical need might bias the analysis. This is why perceived health may be considered as a better indicator of the clinical need for services. Utilisation of three variables – in addition to self-rated general health, breathlessness and depression – makes the adjustment more comprehensive than in previous studies, although the results were quite similar (odds ratios not shown) when these variables were introduced separately into the analyses.

## Conclusion

Several socioeconomic differences, such as variations in income, in quality of housing, in education and even in health, are generally accepted as parts of the 'natural' hierarchy of society. There is, however, wide agreement that inequality in the access to health services is unacceptable. In other words, the principle of horizontal equity in health care delivery implies that people in equal need of care are treated equally. This survey study of the working-age population revealed details regarding the inequity of Finnish health services which were stated in the OECD reports [16,31]. The principal reasons for the inequity may lie in the funding channels and associated structures of the health services.

If the health service structure is taken as given, we may conclude that atypical employment and unemployment mean a decreased burden on the Finnish ambulatory health service system as a whole. However, the burden of public primary health care obviously depends on fluctuations in the unemployment rates and in atypical employment. In order to decrease the inequity of health services, health centres should be provided with adequate resources. Moreover, particular services should be developed, that aim to establish and maintain contact with the most marginal groups in the labour market and meet their specific service needs.

There is no previous research on the use of health services by employees with atypical contracts, and in studies of the unemployed they are commonly merged with either the employed or the unemployed. The present Finnish study should therefore be considered as a starting point for further studies about associations between labour market status and health service use. There are both health policy based and science based reasons for studies comparing internationally the access to health services of employee groups occupying different positions in the core periphery structure of the labour markets.

## Competing interests

The author(s) declare that they have no competing interests.

## Authors' contributions

PV carried out the statistical analyses and was responsible for writing the drafts of the manuscript.

MK and JV participated in designing the study and in the statistical analyses and contributed to the writing of the manuscript.

MK, who coordinated the HeSSup research project, contributed to the design of the study and helped to draft the manuscript.

All authors read and approved the final manuscript

## Pre-publication history

The pre-publication history for this paper can be accessed here:



## Figures and Tables

**Table 1:** Number (per cent) of participants according to labour market status and background variables.

	Labour market status						
	
	Employed, permanent, full-time N = 9 306	Employed, permanent, part-time N = 483	Employed, fixed-term, full-time N = 1 418	Employed, fixed-term, part-time N = 316	Unemployed, short-term N = 683	Unemployed, prolonged N = 222	Unemployed, long-term N = 199
Gender							
Women	5180 (56)	414 (86)	1013 (72)	242 (77)	446 (65)	140 (63)	85 (43)
Men	4118 (44)	68 (14)	404 (28)	74 (23)	236 (35)	82 (37)	114 (57)
Age group							
25–29	1627 (18)	101 (21)	745 (53)	123 (40)	91 (13)	84 (38)	23 (12)
35–39	2349 (25)	149 (31)	345 (24)	69 (22)	108 (16)	48 (22)	43 (22)
45–49	2996 (32)	114 (24)	202 (14)	70 (22)	115 (17)	35 (16)	67 (34)
55–59	2300 (25)	118 (25)	124 (9)	54 (17)	364 (54)	53 (24)	64 (33)
Further education							
None	2029 (22)	154 (33)	455 (33)	137 (44)	274 (42)	92 (43)	99 (51)
Vocational school	2149 (24)	105 (23)	252 (18)	59 (19)	166 (25)	60 (28)	60 (31)
College	3452 (38)	166 (36)	419 (31)	83 (27)	181 (27)	54 (25)	29 (15)
University	1453 (16)	36 (8)	249 (18)	30 (10)	40 (6)	9 (4)	7 (4)
Self-rated health							
Optimal	7595 (82)	353 (74)	1243 (88)	235 (75)	442 (65)	143 (64)	103 (52)
Non-optimal	2640 (18)	126 (26)	166 (12)	77 (25)	240 (35)	79 (36)	95 (48)
Breathlessness							
No	6473 (70)	293 (61)	1002 (71)	201 (65)	391 (57)	132 (60)	101 (51)
Yes	2751 (30)	188 (39)	407 (29)	108 (35)	290 (43)	89 (40)	98 (49)
Depression							
No	7550 (83)	353 (75)	1174 (84)	232 (75)	490 (73)	122 (57)	103 (55)
Yes	1582 (17)	118 (25)	224 (16)	78 (25)	179 (27)	91 (43)	86 (45)
Disease (1)							
None	5362 (47)	226 (37)	938 (54)	158 (42)	250 (32)	92 (36)	64 (9)
One or more	5955 (53)	381 (63)	782 (46)	218 (58)	538 (68)	161 (64)	159 (71)
Cardiorespiratory	2234 (20)	154 (25)	281 (16)	85 (23)	227 (29)	65 (26)	73 (33)
Musculoskeletal	2885 (26)	186 (31)	278 (16)	87 (23)	265 (34)	70 (28)	68 (30)

(1) Self-reported disease diagnosed by a physician.

**Table 2:** Frequency and intensity of physician visits by service provider and labour market status.

Service provider	Labour market status	Visits during the previous twelve months				
		
		None	1	2–4	over 4	Intensity (1)
Public health centre	Employed					
	Permanent full-time	57%	23%	18%	3%	0.91
	Permanent part-time	41%	30%	23%	6%	1.33
	Fixed-term full-time	46%	27%	24%	4%	1.18
	Fixed-term part-time	38%	26%	31%	5%	1.52
	Unemployed					
	Short-term	32%	28%	33%	7%	1.69
	Prolonged	31%	31%	29%	9%	1.71
	Long-term	38%	18%	31%	13%	1.94
Private physician	Employed					
	Permanent full-time	63%	23%	13%	2%	0.73
	Permanent part-time	62%	22%	14%	3%	0.80
	Fixed-term full-time	70%	18%	11%	1%	0.61
	Fixed-term part-time	71%	19%	9%	1%	0.54
	Unemployed					
	Short-term	73%	15%	10%	2%	0.59
	Prolonged	75%	14%	10%	2%	0.55
	Long-term	84%	10%	6%	0%	0.29
Occupational health care	Employed					
	Permanent full-time	42%	25%	27%	6%	1.41
	Permanent part-time	59%	20%	15%	6%	1.03
	Fixed-term full-time	57%	22%	17%	3%	0.94
	Fixed-term part-time	76%	14%	7%	3%	0.54
	Unemployed					
	Short-term	79%	11%	8%	2%	0.47
	Prolonged	91%	4%	3%	2%	0.26
	Long-term	93%	4%	3%	1%	0.16
Hospital outpatient clinic						
	Employed					
	Permanent full-time	74%	14%	10%	2%	0.56
	Permanent part-time	68%	13%	14%	4%	0.80
	Fixed-term full-time	76%	12%	9%	2%	0.53
	Fixed-term part-time	70%	15%	13%	2%	0.66
	Unemployed					
	Short-term	66%	16%	15%	3%	0.78
	Prolonged	64%	15%	17%	5%	0.93
	Long-term	62%	15%	19%	5%	0.99
Dental care	Employed					
	Permanent full-time	33%	36%	26%	5%	1.47
	Permanent part-time	32%	33%	28%	7%	1.57
	Fixed-term full-time	44%	30%	22%	5%	1.22
	Fixed-term part-time	44%	32%	18%	6%	1.21
	Unemployed					
	Short-term	36%	34%	26%	5%	1.39
	Prolonged	42%	30%	23%	5%	1.30
	Long-term	51%	21%	23%	6%	1.22

(1) Mean frequency assuming that class '2–4' = 3 visits and class 'over 4' = 6 visits.

**Table 3:** Coverage and odds ratios for a visit according to labour market status.

Labour market status	Coverage(1)	Adjustments			
		
		None	+ socio-demographics (2)	(2) + perceived health (3)	(2) + disease (4)
Employed					
Permanent full-time	94%	1	1	1	1
Permanent part-time	95%	1.28 (0.85–1.93)	0.99 (0.65–1.52)	0.86 (0.56–1.32)	0.82 (0.57–1.18)
Fixed-term full-time	91%	**0.68 (0.56–0.83)**	**0.67 (0.55–0.83)**	**0.64 (0.51–0.79)**	**0.66 (0.54–0.79)**
Fixed-term part-time	89%	**0.58 (0.43–0.84)**	**0.51 (0.35–0.74)**	**0.46 (0.31–0.68)**	**0.47 (0.33–0.66)**
Unemployed					
Short-term	92%	0.73 (0.48–1.08)	0.69 (0.46–1.04)	**0.60 (0.40–0.91)**	**0.63 (0.43–0.93)**
Prolonged	87%	**0.48 (0.32–0.73)**	**0.47 (0.31–0.71)**	**0.35 (0.23–0.54)**	**0.43 (0.29–0.63)**
Long-term	80%	**0.28 (0.19–0.40)**	**0.29 (0.20–0.43)**	**0.22 (0.15–0.33)**	**0.25 (0.18–0.36)**

(95% confidence intervals without adjustments, with adjustment for socio-demographic factors and with adjustments for health and for disease)

(1) Per cent of respondents with one or more physician visits during the previous twelve months in a public health centre or occupational health care or private services or hospital outpatient clinic or dental services

(2) Gender, age and further education

(3) Self-rated health, breathlessness and depression

(4) Self-reported disease diagnosed by a physician

Interactions with labour market status of the variables included in model (2) + (3):

age, p = 0.103

self-rated health, p = 0.997

sex, p = 0.446

breathlessness, p = 0.499

education, p = 0.398

depression, p = 0.315

**Table 4:** Coverage and odds ratios.

Labour market status	No disease		Disease	
		
	Coverage (1)	Odds ratio	Coverage (1)	Odds ratio
Employed				
Permanent full-time	90%	1	96%	1
Permanent part-time	91%	0.82 (0.50–1.33)	96%	0.84 (0.48–1.47)
Fixed-term full-time	88%	**0.66 (0.52–0.83)**	94%	**0.65 (0.46–0.91)**
Fixed-term part-time	84%	**0.45 (0.29–0.70)**	93%	**0.51 (0.29–0.88)**
Unemployed				
Short-term	84%	**0.49 (0.31–0.79)**	96%	0.98 (0.48–2.03)
Prolonged	76%	**0.31 (0.19–0.51)**	94%	0.67 (0.33–1.35)
Long-term	67%	**0.23 (0.13–0.39)**	86%	**0.27 (0.17–0.44)**

Coverage and odds ratios (95% confidence intervals adjusted for age, sex and further education) for a physician visit according to labour market status in participants without and with self-reported disease diagnosed by a physician.

(1) Per cent of respondents with one or more physician visits during the previous twelve months in a public health centre or occupational health care or private services or hospital outpatient clinic or dental services

**Table 5:** Coverage and odds ratios.

	Public health centre		Occupational health care		Private services	
			
Labour market status	Coverage (1)	Odds ratio	Coverage (1)	Odds ratio	Coverage (1)	Odds ratio
Employed						
Permanent full-time	43%	1	58%	1	37%	1
Permanent part-time	59%	**1.56 (1.27–1.91)**	41%	**0.45 (0.37–0.55)**	38%	0.83 (0.67–1.02)
Fixed-term full-time	54%	**1.47 (1.30–1.67)**	42%	**0.52 (0.46–0.59)**	30%	**0.73 (0.64–0.84)**
Fixed-term part-time	62%	**1.74 (1.36–2.23)**	24%	**0.22 (0.17–0.29)**	29%	**0.62 (0.45–0.81)**
Unemployed						
Short-term	68%	**2.34 (1.81–3.03)**	23%	**0.22 (0.17–0.29)**	27%	**0.59 (0.45–0.77)**
Prolonged	69%	**2.16 (1.59–2.95)**	9%	**0.06 (0.04–0.10)**	25%	**0.52 (0.37–0.74)**
Long-term	62%	**1.63 (1.19–2.25)**	7%	**0.05 (0.03–0.08)**	16%	**0.32 (0.21–0.49)**

	Hospital outpatient clinic		Dental care			
				
Labour market status	Coverage (1)	Odds ratio	Coverage (1)	Odds ratio		
		
Employed						
Permanent full-time	26%	1	66%	1		
Permanent part-time	34%	1.06 (0.85–1.32)	65%	0.84 (0.68–1.03)		
Fixed-term full-time	24%	0.90 (0.78–1.04)	56%	**0.77 (0.68–0.88)**		
Fixed-term part-time	30%	1.04 (0.79–1.36)	55%	**0.69 (0.54–0.88)**		
Unemployed						
Short-term	36%	1.27 (0.97–1.65)	62%	0.92 (0.72–1.18)		
Prolonged	36%	1.13 (0.83–1.55)	57%	0.75 (0.56–1.01)		
Long-term	39%	1.20 (0.86–1.68)	48%	**0.47 (0.34–0.64)**		

Coverage and odds ratios (95 % confidence intervals adjusted for gender, age, further education, self-rated health, breathlessness and depression) for a visit according to labour market status and service sector.

(1) Per cent of respondents with one or more physician visits during the previous twelve months

**Table 6:** Coverage and odds ratios.

	Cardiorespiratory disease				Musculoskeletal disease			
		
Labour market status	Primary health care		Hospital outpatient clinic		Primary health care		Hospital outpatient clinic	
				
	Coverage (1)	Odds ratio	Coverage (1)	Odds ratio	Coverage (1)	Odds ratio	Coverage (1)	Odds ratio
Employed								
Permanent full-time	93%	1	37%	1	91%	1	36%	1
Permanent part-time	94%	0.89 (0.44–1.79)	47%	1.36 (0.96–1.94)	89%	0.69 (0.42–1.15)	43%	1.25 (0.91–1.72)
Fixed-term full-time	85%	**0.46 (0.31–0.67)**	37%	1.00 (0.76–1.32)	89%	0.77 (0.51–1.16)	38%	1.07 (0.82–1.40)
ixed-term part-time	85%	**0.38 (0.20–0.73)**	40%	1.06 (0.66–1.71)	84%	**0.49 (0.27–0.91)**	44%	1.33 (0.85–2.09)
Unemployed								
Short-term	92%	0.71 (0.32–1.60)	43%	1.26 (0.78–2.03)	84%	**0.46 (0.34–0.88)**	51%	1.49 (0.92–2.42)
Prolonged	83%	**0.33 (0.17–0.65)**	48%	1.51 (0.90–2.53)	94%	1.50 (0.54–4.17)	45%	1.33 (0.80–2.20)
Long-term	85%	**0.38 (0.19–0.75)**	50%	1.57 (0.96–2.58)	81%	**0.48 (0.25–0.92)**	55%	**2.27 (1.37–3.76)**

Coverage and odds ratios (95 % confidence intervals adjusted for gender, age and further education) for a visit in primary health care (public health centre or occupational health care or private services) and in hospital outpatient clinic according to labour market status in participants having a cardiorespiratory disease and in participants having a musculoskeletal disease diagnosed by a physician.

(1) Per cent of respondents with one or more physician visits during the previous twelve months

## References

[B1] Iversen L, Sabroe S, Damsgaard M (1989). Hospital admissions before and after shipyard closure. BMJ.

[B2] Beale N, Nethercott S (1988). The nature of unemployment morbidity; 2. description. J Coll Gen Pract.

[B3] Studnicka M, Studnicka-Benke A, Wogerbauer G, Rastetter D, Wenda R, Gathmann P, Ringel E (1991). Psychological health, self-reported physical health and health service use. Risk differential observed after one year of unemployment. Soc Psych and Psych Epidemiol.

[B4] Virtanen P (1993). Unemployment, re-employment and the use of primary health care services. Scand J Prim Health Care.

[B5] Studnicka M, Schreiber V (1988). Arbeitslosigkeit und Inanspruchnahme des Gesundheitssystems [Unemployment and utilization of the public health system]. Offentliche Gesundheitswesen.

[B6] D'Arcy C, Siddique C (1985). Unemployment and health: an analysis of "Canada Health Survey" data. Int J Health Serv.

[B7] Kraut A, Mustard C, Walld R, Tate R (2000). Unemployment and health care utilization. Scand J Work Environ Health.

[B8] Yuen P, Balarajan R (1989). Unemployment and patterns of consultation with the general practitioner. BMJ.

[B9] Kontula O, Viinamäki H, Koskela K (1998). Köyhiä ja kipeitä: [The poor and the sick].

[B10] Ferrie J, Marmot M, Griffiths J, Ziglio E, (Eds) (1999). Labour market changes and job insecurity: a challenge for social welfare and health promotion.

[B11] Isaksson K, Hogstedt C, Eriksson C, Theorell T, (Eds) (2000). Health Effects of the New Labour Market.

[B12] Zeytinoglu I, (Ed) (2002). Flexible work arrangements: conceptualizations and international experiences.

[B13] Virtanen P, Liukkonen V, Vahtera J, Kivimäki M, Koskenvuo M (2003). Health inequalities in the work force: the labour market core-periphery structure. Int J Epidemiol.

[B14] Kasl S, Gore S, Kobb S (1975). Experience of losing a job: reported changes in health, symptoms and illness behavior. Psychosom Med.

[B15] Bartley M, Fagin L (1990). Hospital admissions before and after shipyard closure. Br J Psychiatry.

[B16] van Doorslaer E, Masseria C (2004). Income-related inequality in the use of medical care in 21 OECD countries. OECD Social Issues/Migration/Health.

[B17] Smaje C, Le Grand J (1997). Ethnicity, equity and the use of health services in the British NHS. Soc Sci Med.

[B18] Fernandez de la Hoz K, Leon D (1996). Self perceived health status and inequalities in use of health services in Spain. Int J Epid.

[B19] Hjern A, Haglund B, Persson G, Rosen M (2001). Is there equity in access to health services for ethnic minorities in Sweden?. Eur J Public Health.

[B20] Stronks K, Ravelli A, Reijneveld S (2001). Immigrants in the Netherlands: equal access for equal needs?. J Epidemiol Comm Health.

[B21] Beck A, Ward C, Mendelson M, Mock J, Erbaugh J (1961). An inventory for measuring depression. Arch Gen Psych.

[B22] Häkkinen U (2005). Change in determinants of use of physician services in Finland between 1987 and 1996. Soc Sci Med.

[B23] Hetemaa T, Keskimäki I, Salomaa V, Mähönen M, Manderbacka K, Koskinen S (2004). Socioeconomic inequities in invasive cardiac procedures after first myocardial infarction in Finland in 1995. J Clin Epidmiol.

[B24] Virtanen M, Kivimäki M, Elovainio M, Vahtera J, Cooper C (2001). Contingent employment, health and sickness absence. Scand J Work Environ Health.

[B25] Zola I (1972). Medicine as an institution of social control. Sociol Review.

[B26] Foucault M (1981). Power/knowledge, selected interviews and other writings 1972–1977.

[B27] Ritter P, Stewart A, Kaymaz H, Sobel D, Block D, Lorig K (2001). Self-reports of health care utilization compared to provider records. J Clin Epidemiol.

[B28] Korkeila K, Suominen S, Ahvenainen J, Ojanlatva A, Rautava P, Helenius H, Koskenvuo M (2001). Non-response and related factors in a nation-wide health survey. Eur J Epidemiol.

[B29] Statistics Finland (1999). Työvoimatilasto 2003 (Labour force statistics 2003).

[B30] Sutton M, Carr-Hill R, Gravelle H, Rice N (1999). Do measures of self-reported morbidity bias the estimation of determinants of health care utilisation?. Soc Sci Med.

[B31] (2005). OECD Reviews of Health Systems – Finland OECD.

